# Metadynamics simulations leveraged by statistical analyses and artificial intelligence-based tools to inform the discovery of G protein-coupled receptor ligands

**DOI:** 10.3389/fendo.2022.1099715

**Published:** 2022-12-23

**Authors:** Leslie Salas-Estrada, Bianca Fiorillo, Marta Filizola

**Affiliations:** Department of Pharmacological Sciences, Icahn School of Medicine at Mount Sinai, New York, NY, United States

**Keywords:** GPCRs (G protein-coupled receptors), metadynamics, molecular dynamics simulation, machine learning, enhanced sampling

## Abstract

G Protein-Coupled Receptors (GPCRs) are a large family of membrane proteins with pluridimensional signaling profiles. They undergo ligand-specific conformational changes, which in turn lead to the differential activation of intracellular signaling proteins and the consequent triggering of a variety of biological responses. This conformational plasticity directly impacts our understanding of GPCR signaling and therapeutic implications, as do ligand-specific kinetic differences in GPCR-induced transducer activation/coupling or GPCR-transducer complex stability. High-resolution experimental structures of ligand-bound GPCRs in the presence or absence of interacting transducers provide important, yet limited, insights into the highly dynamic process of ligand-induced activation or inhibition of these receptors. We and others have complemented these studies with computational strategies aimed at characterizing increasingly accurate metastable conformations of GPCRs using a combination of metadynamics simulations, state-of-the-art algorithms for statistical analyses of simulation data, and artificial intelligence-based tools. This minireview provides an overview of these approaches as well as lessons learned from them towards the identification of conformational states that may be difficult or even impossible to characterize experimentally and yet important to discover new GPCR ligands.

## Introduction

G Protein-Coupled Receptors (GPCRs) are important drug targets consisting of seven membrane-spanning helices connected by alternating intracellular and extracellular loops, and known for transducing extracellular signals across the cell membrane. Evidence accumulated over the past decade has suggested a pluridimensional functionality of these receptors induced by ligands with different efficacies. Specifically, ligands as diverse as photons, small molecules, and peptides can stabilize different receptor conformations which, in turn, can trigger the activation of different effectors, such as several subtypes of heterotrimeric G proteins, β-arrestins, and G protein-coupled receptor kinases (1). This differential engagement of transducer subtypes with varying signal magnitudes is a phenomenon known as “functional selectivity” or “biased agonism” (2–7), and is a prerequisite for triggering therapeutic or unwanted biological effects of GPCRs *via* activation of different downstream signaling pathways. However, it is not the only element at play. Temporal analyses of ligand binding to GPCRs, as well as GPCR-induced G protein activation/coupling and β-arrestin recruitment evoked by ligands with different efficacies, demonstrate the existence of another dimension of functional bias by GPCRs that directly impacts our understanding of GPCR signaling and therapeutic implications, thus suggesting the importance of incorporating quantifications of ligand binding and signaling kinetics in modern drug discovery efforts (8–10).

The mechanistic and kinetic bases of GPCR-mediated functional selectivity are poorly understood, notwithstanding the past decade’s technological advances in GPCR functional and structural biology. Among them are the availability of genetically-encoded biosensors for the optical detection of signals from a variety of transduction molecules in several cell types, tissues, and whole organisms (11), as well as revolutionary methodological developments in X-ray crystallography and cryogenic electron microscopy (cryo-EM). The latter have allowed to solve high-resolution experimental structures for 140 unique GPCRs, 520 unique ligand-GPCR complexes, 95 unique GPCRs in complex with heterotrimeric G proteins, 6 unique GPCRs in complex with arrestins, and 1 unique GPCR in complex with G protein-coupled receptor kinases (data retrieved from GPCRdb (12) and the Protein Data Bank (13) on 11/4/2022). Although these structures have provided important insights into ligand-GPCR and GPCR-transducer interactions, they are heavily engineered static snapshots. Thus, inferences of long-distance conformational changes propagating from the ligand binding site in the receptor to its cellular signaling partners, and eventually translating into specific physiological cell responses, remain highly speculative.

**Figure 1:**
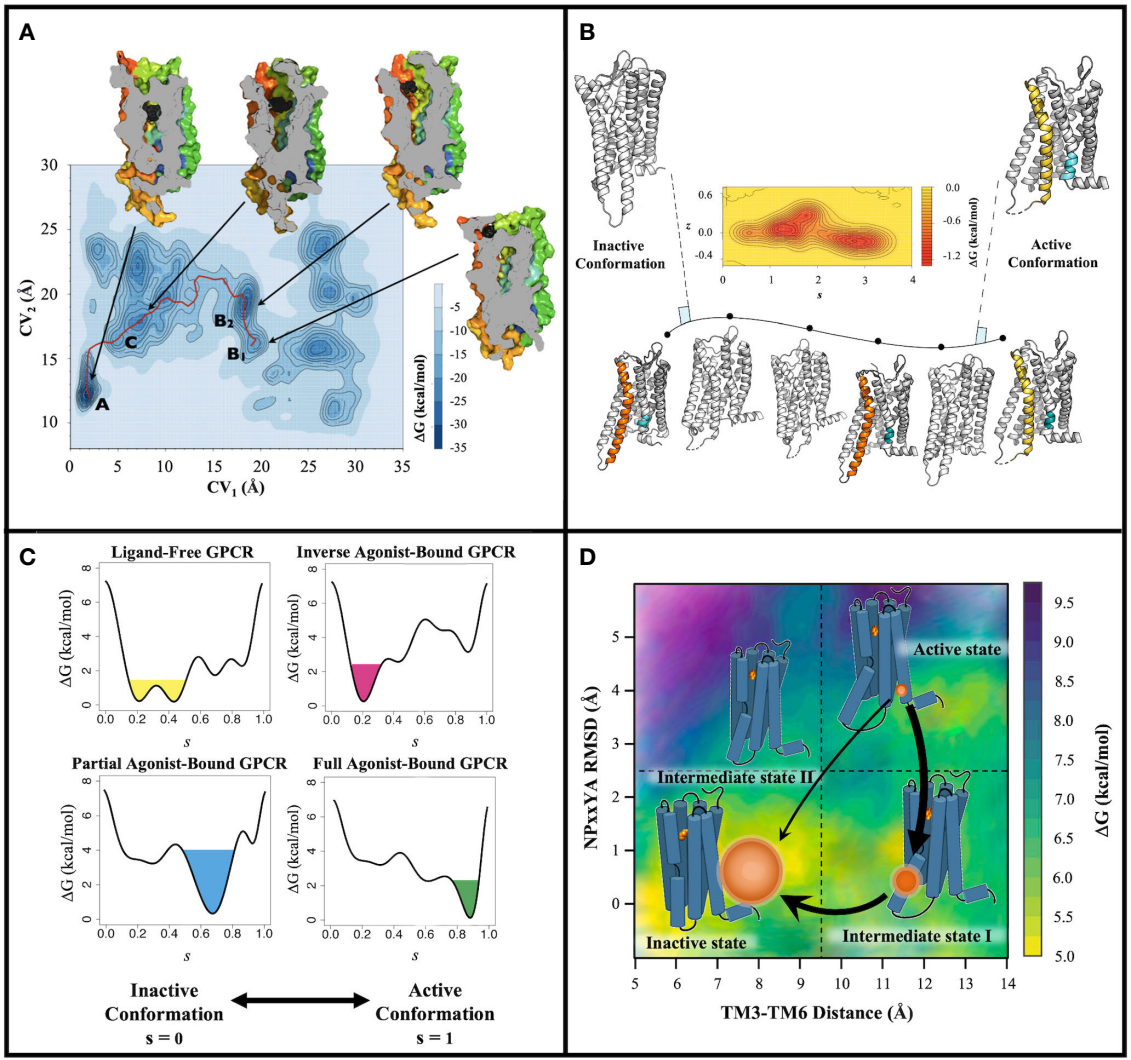
Examples of output information from MetaD-based strategies adapted to GPCRs. **(A)** Illustration of output information (free-energy surface and low-energy conformational states) from a MetaD-based strategy aimed at efficiently exploring molecular mechanisms of GPCR ligand recognition. Specifically, the free-energy surface is reconstructed as a function of the distance of the ligand’s center of mass (CV_1_) and of the distance of the extracellular loop 2’s center of mass (CV_2_) from the center of mass of the receptor binding pocket. Relevant states along the binding pathway are labeled A, B_1_, B_2_, and C The red solid line refers to the proposed entry path of the ligand. Also represented are images of A, B_1_, B_2_, and C metastable states of the receptor cut along their TM4 face as well as the position of the ligand (black spheres) in the corresponding states. Reprinted with permission from Provasi, D., Bortolato, A., Filizola, M. “Exploring Molecular Mechanisms of Ligand Recognition by Opioid Receptors with Metadynamics” Biochemistry (2009), 48(42): 10020–10029, Copyright ^©^ 2009, American Chemical Society (31); **(B)** Illustration of the combined adiabatic biased MD and path-sampling MetaD strategy used to characterize GPCR activation energy landscapes. The free energy between inactive and active conformations is reconstructed as a function of the position along the path (*s*) and the distance (*z*) from it. **(C)** Example of ligand-induced modulation of the free-energy landscape of a GPCR as a function of the position (s) along the activation pathway. **(D)** Illustration of thermodynamic and kinetic information derived from a combination of path collective variables MetaD simulations and the maximum caliber principle.

To probe the conformational heterogeneity of GPCRs and GPCR complexes and extend information from high-resolution structural methods, researchers have resorted to biophysical techniques such as nuclear magnetic resonance (14–21), double electron-electron resonance spectroscopy (22), hydrogen/deuterium exchange mass spectroscopy (23, 24), and single-molecule fluorescence resonance energy transfer (25–27). However, current technical challenges prevent these techniques from achieving atomic-level precision for the entire GPCR alone or in complex with their natural cellular signaling partners. Although molecular dynamics (MD) simulations can provide a critical bridge between the atomic-level insight from high-resolution structural methods and molecular motions (28), standard MD algorithms limit dynamic explorations to timescales that are shorter than most biological processes notwithstanding their use of massively-parallel high-performance computing platforms. Several enhanced conformational sampling methods have been put forward to overcome these limitations (29), and our group pioneered the use of one of them, *i.e.*, metadynamics (MetaD) [see **Box 1** and (30)], for more efficient studies of the conformational plasticity of liganded or unliganded GPCRs embedded in a lipid mimetic environment. The underlying principles of MetaD are that (a) the process to be investigated can be described by a small number of reaction coordinates (collective variables, CVs) and (b) the sum of destabilizing Gaussian potentials that are added to penalize sampled conformational states for faster simulation convergence is the mirror image of the free-energy profile of said process. Although MetaD’s main problem remains that of identifying the right CVs to efficiently study the dynamic process of interest, MetaD-based strategies are increasingly utilized nowadays to study GPCR ligand binding, conformational dynamics, and kinetics (see **Figure 1** for illustrations of selected strategies), thus offering an outlet where one could exploit uniquely characterized metastable states of GPCRs as targets for the discovery of functionally selective ligands.

Box 1 Metadynamics is a powerful enhanced sampling method introduced by Alessandro Laio and Michele Parrinello in 2002 to efficiently study the slow dynamics of complex systems. It extends the utility of classical molecular dynamics simulations by adding a time-dependent bias potential to the system’s Hamiltonian to enable the exploration of larger portions of its phase space within a given amount of simulation time. This bias potential is constructed over time as a sum of local repulsive potential terms (e.g., Gaussian functions) centered at the sampled values of reaction coordinates (or collective variables) that are appropriately chosen to describe the system’s slow dynamics. As a result, the system is encouraged to sample unexplored regions of its phase space, yielding faster convergence and a thorough free energy landscape that can be reconstructed from the bias. Although identifying effective collective variables remains a challenging problem, several variants of the original algorithm were developed over the past two decades to address some of its main limitations and are increasingly utilized nowadays to study slow processes of complex biological systems, including GPCR ligand binding, conformational dynamics, and kinetics.

This minireview provides a general overview of MetaD strategies used alone or in combination with algorithms for statistical analyses of simulation data [*e.g.*, Markov State Models (MSMs) (32, 33), information theory-based methods (34), and transfer entropy approaches (35)] and machine learning/artificial intelligence (AI)-based tools (36) to not only characterize ligand-specific conformational states of GPCRs that are difficult to resolve experimentally but also to derive information that can help expedite the GPCR drug discovery process. This includes structural determinants of allosteric communications from the causality of correlated motions, as well as ligand-specific kinetic elements of activation [e.g., (37, 38)]. The computational strategies and applications discussed herein are not exhaustive but include representative examples that have affirmed the power of MetaD in the study of various GPCR processes, including ligand binding, receptor activation, and related kinetics. The different simulation setups and protocols of the studies reported in this minireview are summarized in **Table 1**.

**Table 1:** Summary of the simulation setups and protocols of the MetaD studies reported in the main text.

	GPCR	MetaD Variant	CVs (#, type)	System setup	Size (# atoms)	Simul. time (μs)	Force field	Ref.
Ligand Binding	β_2_AR MOR M_2_R V_1A_R V_2_R	FM, MW-, WT-MetaD	1, Trp^6.48^-ligand distance	ANT/AGO+β_2_AR (2RH1/3SN6)-Gαs/none, AGO+MOR (5C1M)-Nb/Gαi/none, AGO+M_2_R(4MQS)-Nb/Gαi/none, ANT/PART AGO+V_1A_R (model), ANT/PART AGO+V_2_R (model) DOPC	NR	0.5-2.2	AMBERff99SB-ILDN/GAFF/AM1-BCC	(39)
	β_2_AR H_1_R	FM, MW-, WT-MetaD	1, Trp^6.48^-ligand distance	ANT/AGO+β_2_AR(4LDO), AGO+ H_1_R(3RZE) DOPC	~98K	1.5	AMBERff99SB/GAFF/AM1-BCC	(40)
	DOR	MW-, WT-MetaD	2, ligand- and EL2-binding pocket distance	ANT+DOR(model) DPPC/CHOL (8:2)	~47K	0.5	OPLS-AA/ Berger united-atom lipid parameters	(31)
	DOR	MW-MetaD	2, receptor-ligand distance and # contacts	PAM+DOR(4N6H)-AGO POPC/CHOL (9:1)	~78K	~3.6	CHARMM36m/CGenFF	(41)
	MOR	MW-, WT-MetaD	2, receptor-ligand distance and # contacts	AGO+MOR(4DKL) POPC/CHOL (9:1)	~58K	6.5-7.0	CHARMM36/ CGenFF	(42)
	MOR	BPMD	1, ligand RMSD w.r.t. docked pose	AGO+MOR(5C1M) POPC	NR	0.1	OPLS3e/Force Field Builder	(43)
	MOR	BPMD	1, ligand RMSD w.r.t. docked pose	AGO+MOR(5C1M)-Nb POPC/CHOL (9:1)	~61K	0.1	CHARMM36/ CGenFF	(44)
Receptor Activation and Ligand-Specific Conformations	5-HT_1B_R 5-HT_2A_R 5-HT_2C_R α_2A_AR β_2_AR A_1_R A_2A_R CB_1_ CCR5 D_2_R DOR KOR MOR M_1_R M_3_R	MW-, WT-MetaD	2, TM3-TM6 distances 2, TM3- and TM7-TM6 distances 2, distances in GP 2, TM3-TM6 and GP distances 3, GP, TM3- and TM7-TM6 distances 3, TM3/TM6/TM5-GP distances 3, TM3/TM6-GP distances and TM3-TM6 distance 3, TM3-GP distances and TM3-TM6 distance 3, distances in GP	5-HT_2A_R(6WH4/ 6WHA)- INV AGO/PART AGO/ FULL AGO/none-G(model)/Gαq peptide, 5-HT_1B_R(5V54)-Go(6G79), 5-HT_2C_R(6BQH)-Gq(6WHA), α_2A_AR(6KUX)-Gq(model), β_2_AR(2RH1)-T4L/Gs(3SN6)/none, A_1_R(5UEN)-Gi2(6D9H), A_2A_R(6GDG/3EML)-AGO/none-Gs(3SN6), CB_1_(5TGZ)-Gi1(6N4B), CCR5(5UIW)-Gi1(6DDF), D_2_R(6LUQ)-Go(6VMS), DOR(4N6H)-Gi1(6DDF), KOR(4DJH)-Gi1(GDDF), MOR-Gi1, M_1_R(6WJC)-G11(6OIJ), M_3_R(5ZHP)-Gq(model) POPC or POPC:CHOL (4:1) for A_2A_R-AGO-Gs	49K-230K	0.2-2.5	CHARMM36m/CGenFF	(45)
	β_2_AR	PS-, WT-MetaD	2, position along and distance from ABMD-derived activation pathway	β_2_AR(2RH1)-INV AGO/ANT/PART AGO/FULL AGO/none POPC/CHOL (9:1)	~50K	0.3	OPLS-AA/ Berger united-atom lipid parameters	(46)
	β_2_AR	FM, WT-MetaD	1, Trp^6.48^-ligand distance 1, TM3-TM6 distance 1, TM3-Gs/β-arrestin distance	β_2_AR(2RH1/3SN6)-INV AGO/ANT/AGO-Gs(3SN6)/β-arrestin (4JQI/4ZWJ)/none DOPC	NR	2.0-7.0	AMBERff99SB-ILDN/ GAFF/AM1-BCC	(47)
	A_2A_R	WT-MetaD	2, Trp^6.48^ side chain dihedrals	A_2A_R(2YDO/2YDV/3QAK/3EML/3PWH/3REY/3RFM)-INV AGO/AGO/none POPC	~65K	0.1	CHARMM36-lipids/ CHARMM27-cmap CGenFF	(48)
	GABA_B_R	WT-MetaD	1, TM3-Gi distance 1, TM4-TM5 distance 1, distance in Gi	GABA_B_R(7C7Q/7EB2/6UO9/SF from 7EB2 system)-AGO-Gi(model)/none POPC/CHOL (37:1)	220K-490K	~0.2	CHARMM36m/CGenFF	(49)
	GCGR	PT-, MW-, WT-MetaD	2, RMSD_GLP-1R*_ ± RMSD_GCGR_ of TM6 Cαs 1, receptor-Gαs distance	GCGR(5YQZ)-PART AGO-Gαs(6EG8)/none DOPC	NR	4.0-12.7	AMBER14SB/ LipidBook	(50)
	MOR	PS-, WT-MetaD	2, position along and distance from ABMD-derived activation pathway	MOR(4DKL/5C1M)-AGO POPC/CHOL (9:1)	~54K	3.0	CHARMM36/ CGenFF	(38)
	RHO	PS-, WT-MetaD	2, position along and distance from ABMD-derived activation pathway	RHO(2I37)-AGO POPC	~45K	0.24	OPLS-AA/ Berger united-atom lipid parameters	(51)
Binding and Activation Kinetics	A_2A_R	SuMetaD	2, position and distance along binding pathway	ANT+A_2A_R(4EIY/3REY) DMPC	NR	0.01	AMBER99SB/ GAFF/AM1-BCC	(52)
	MOR	In-, WT-MetaD	2, ligand hydration and ML-optimized RC	AGO+MOR(5C1M) POPC/CHOL (9:1)	NR	~15	CHARMM36/ CGenFF	(37)
	MOR	WT-MetaD	2, receptor-ligand distance and # contacts	AGO+MOR(5C1M/most populated conf. from (53)) POPC/CHOL	~79K	2.0-4.0	CHARMM36m/CGenFF	(54)

5-HT_1B_R, 5-hydroxytryptamine receptor 1B; 5-HT_2A_R, 5-hydroxytryptamine receptor 2A; 5-HT_2C_R, 5-hydroxytryptamine receptor 2C; α_2A_AR, α_2A_-adrenergic receptor; β_2_AR, β_2_-adrenergic receptor; A_1_R, adenosine A_1_ receptor; A_2A_R, adenosine A_2A_ receptor; ABMD, adiabatic biased molecular dynamics; BE-MetaD, bias-exchange metadynamics; AGO, agonist; ANT, antagonist; BPMD, binding pose metadynamics; CB_1_, CB_1_ cannabinoid receptor; CCR5, CC-chemokine receptor 5; CGenFF, CHARMM General Force Field; CHOL, cholesterol; COM, center of mass; CVs, collective variables; DOR, δ-opioid receptor; D_2_R, dopamine D_2_ receptor; DMPC, 1,2-dimyristoyl-sn-glycero-3-phosphocholine; DOPC, 1,2-Dioleoyl-sn-glycero-3-phosphocholine; DPPC, 1,2-dipalmitoyl-sn-glycero-3-phosphocholine; EL2, extracellular loop 2; FM, funnel-metadynamics; FMAP, funnel-metadynamics advanced protocol; FULL AGO, full agonist; GABA_B_R, γ-aminobutyric acid(B) receptor; GAFF, generalized AMBER force field; GCGR, glucagon receptor; GLP1R, glucagon-like peptide 1 receptor; GP, G protein; H_1_R, histamine H_1_ receptor; In-MetaD, infrequent metadynamics; INV AGO, inverse agonist; KOR, κ-opioid receptor; MOR, μ-opioid receptor; M_1_R, muscarinic M_1_ receptor; M_3_R, muscarinic M_3_ receptor; MW-MetaD, multiple-walker metadynamics; Nb, nanobody; NR, not reported; PAM, positive allosteric modulator; PART AGO, partial agonist; PS-MetaD, path-sampling metadynamics; PT-MetaD, parallel tempering metadynamics; POPC, 1-palmitoyl-2-oleoyl-sn-glycero-3-phosphocholine; RC, reaction coordinate; RHO, rhodopsin; RMSD, root mean squared deviation; SF, simulation frame; SuMetaD, supervised metadynamics; T4L, T4 lysozyme; TM, transmembrane.

## MetaD-based strategies for the prediction of GPCR-ligand binding modes

To the best of our knowledge, the very first application of MetaD to GPCRs was published by our group more than a decade ago (31) and aimed at efficiently exploring the molecular mechanisms of GPCR ligand recognition (see **Figure 1A** for an example of output information). Specifically, we studied the free binding of the antagonist naloxone from the water environment to a homology model of the δ-opioid receptor (DOR) based on the X-ray crystal structure of the β_2_-adrenergic receptor (β_2_AR) as there were no available crystal structures of opioid receptors at the time (see **Table 1** for details of system setup and simulations). Although one would have ideally used a single CV to contain the computational cost, which scales exponentially with the number of CVs (55), we could not achieve simulation convergence for this system with less than two CVs and a half microsecond of well-tempered (WT) MetaD simulations using multiple walkers (MW) (56). These simulations revealed, for the first time, unprecedented details of the free binding of a ligand to a GPCR, including the various intermediate energetic states visited by the ligand and their corresponding ligand-receptor interactions. Moreover, by restricting ligand sampling in the bulk region using an approach that allowed the ligand to only move in a conical region centered at the center of mass (COM) of the binding pocket (57), and applying the appropriate correction to the calculated free energy, we could derive ligand binding affinity estimates that were close to experimental values (58–61).

Subsequent applications by our group focused on predicting optimal ligand binding modes for atypical opioid ligands (42), as well as opioid allosteric modulators (41), which would be targeting regions on the receptor that are not conserved and highly flexible. Ligand binding in these studies was described by CVs accounting for the relative position and orientation of the ligands, as well as the number of interactions they established with the receptor. Although these simulations allowed for a thorough and efficient exploration of the dynamic process of ligand-GPCR binding at either orthosteric or allosteric sites (62, 63), they were computationally quite expensive as they required up to 7 μs of simulation time to reach convergence (42).

To ensure faster simulation convergence, a universal single CV that uses the highly conserved Trp^6.48^ [superscript refers to the Ballesteros-Weinstein numbering scheme for GPCRs (64)] at the base of the orthosteric binding pocket and the orientation of the receptor in the membrane was proposed for ligand binding/unbinding to/from class A GPCRs using WT-MetaD and a funnel restraint (65) that limits the conformational sampling of the ligand in the bulk water (39) (**Table 1**). Funnel MetaD (FM) has been successfully applied to a number of GPCR systems (62), and its use made more easily accessible by a recently reported graphical user interface (GUI)-based protocol termed FM Advanced Protocol (FMAP) (66). Also interesting is a protocol that has recently enabled the successful prediction of preferential binding modes of different GPCR systems using conformational ensembles derived from the clustering of MW-MetaD simulations (40). These strategies, however, are still not amenable for high-throughput given their computational cost. If estimates of protein-ligand binding free energies are not required but the goal is focused on assessing the relative stability of binding mode predictions, a reasonable alternative we and others have used in applications to GPCRs [e.g., (43) for a recent example from our lab] is a combination of induced-fit docking and MetaD (67). Based on these growing examples and the ready accessibility of these strategies *via* user-friendly graphical interfaces, we expect that MetaD-based approaches will become a standard tool for the prediction of GPCR ligand binding in the future, and will make a real impact on drug discovery.

## MetaD-based strategies to probe the activation landscape of GPCRs

We also were the first to study the activation pathway of a GPCR, specifically rhodopsin (RHO), using a combination of MetaD with adiabatic biased MD simulations (51). In particular, we used the MetaD variant known as path-sampling MetaD (PS-MetaD) to reconstruct the system’s free-energy landscape along predetermined transition trajectories between receptor inactive and active states as a function of the position along the path (*s*) and the distance (*z*) from it (**Figure 1B**). Our results suggested that at least four metastable macrostates containing receptor conformations with a different amplitude of the outward movement of transmembrane (TM) helix 6 are sampled by RHO in the transition from inactive to active conformations and these are connected by at least two different pathways (51). The conformations of two of these macrostates were very close to the available inactive and active experimental structures of RHO, whereas the other two macrostates contained conformations representing intermediate states. Notably, subsequent MetaD simulations we carried out on the β_2_AR (46) (see section below for additional details and **Table 1**) confirmed the presence of one different inactive-like and one different active-like macrostates in addition to those whose conformations closely resembled the experimentally known inactive and active structures of the receptor. This finding was interesting since standard MD simulations of the β_2_AR at the time had only been able to identify one intermediate state in terms of TM3-TM6 separation (68). Similar results were also obtained for the μ-opioid receptor (MOR) by carrying out adiabatic biased MD simulations and PS-MetaD simulations (38), as well as more expensive adaptive-sampling MD simulations (44). In contrast, only one intermediate state between fully inactive and active conformations was identified for the class B glucagon receptor (GCGR) (50), using parallel tempering WT-MetaD and CVs representing two linear combinations of the alpha-carbon root mean square deviation (RMSD) of TM6 to the inactive conformation of GCGR and to the active, closely related, glucagon-like peptide 1 receptor (69, 70). An interesting observation of this study based on a comparison between the computed conformational free-energy landscape associated with the activation of the receptor-agonist complex and that of the receptor-agonist-G protein complex was that the agonist stabilizes the receptor in a preactivated complex before its full activation is achieved by G protein binding (50).

MetaD was also recently used to propose the activation mechanisms of several prototypic class A (45) and class C (49) receptors. These mechanisms were class-specific and revealed an active role of the G protein in promoting conformational changes of the receptor.

## MetaD-based strategies for the characterization of ligand-specific GPCR conformations

Being able to predict the receptor conformations a drug can stabilize is considered the “holy grail” in the drug discovery field as it might inform the drug’s biological outcome. With this in mind, we developed a computational strategy to study the ligand-induced modulation of the free-energy landscape of GPCRs (46) (see **Figure 1C** for an example of output information). Specifically, we used WT-MetaD  (56) to identify ligand-specific metastable states of the β_2_AR, along pre-determined activation pathways between its high-resolution inactive and active structures using adiabatic biased MD (**Table 1**). The results confirmed a tendency of ligands to stabilize an inactive or active conformation of the receptor depending on their efficacy (46).

That ligands with different efficacies shift the equilibrium between inactive and active states was recently also reported for the human adenosine A2A receptor using MetaD (48). Specific conformational rearrangements of key structural elements were responsible for this shift, including rotameric changes of the conserved Trp^6.48^ residue. This movement can be induced by agonists but not inverse agonists, and appears to correlate with the opening of the G protein binding site *via* disruption of hydrophobic packing which causes TM6 and TM5 to move away from TM3.

MetaD was also recently used to study the interplay between GPCR ligand binding and the coupling of arrestins or G proteins (47). In addition to estimate the binding free energies of ligands with different efficacies at the β_2_AR in the presence or absence of Gs or β-arrestin, a combination of WT-MetaD and FM was used to study the free energy landscape of activation and transducer coupling as a function of the distance between the receptor alpha-carbons of Arg^3.50^ and Leu^6.34^ and the distance between Arg^3.50^ and the alpha-carbons of Gαs Glu392 or β-arrestin Val71. This setup (**Table 1**) allowed to quantify the ligand/transducer cooperative effects on the activation of the β_2_AR, thus providing a simple way to predict the functional bias of a ligand (47).

## MetaD-based strategies for the prediction of ligand binding and activation kinetics

Knowledge of GPCR ligand binding kinetics, and especially drug-target residence times and involved molecular determinants, is highly desirable because it can serve as an effective guiding principle to select candidate molecules for clinical development (10). However, this collective information is difficult to obtain because on and off rates depend on the height of the highest energy barrier of the transition state between bound and unbound conformations, and this height is difficult to estimate both experimentally and computationally. MD simulations can help study ligand binding at the active site, but binding is a rare event on microscopic timescales, and as such, it is difficult to sample. Although microsecond-scale MD simulations allow to obtain multiple binding events at a GPCR embedded in an explicit lipid-water environment, from which it is possible to derive k_on_ estimates (71), much longer timescales would be required for the dissociation of the ligand from a GPCR orthosteric site, making it very difficult to derive k_off_ estimates from unbiased, standard MD simulations. To attempt more efficient predictions of dissociation rates from a GPCR, we recently designed a computational strategy that combines machine learning and infrequent MetaD (37). Specifically, the strategy used the automatic mutual information noise omission (AMINO) algorithm (72) to enable the robust and automated selection of non-redundant molecular features from unbiased MD simulations that can then be used in the reweighted autoencoded variational Bayes for enhanced sampling (RAVE) method (73, 74) to learn optimal CVs. These CVs were used in infrequent MetaD simulations and allowed to efficiently study the unbinding kinetics of two opioid drugs with very different residence times (morphine and buprenorphine) and to predict dissociation rates for these drugs from the MOR that were within one order of magnitude from experimental values (75). The simulations also gave structural information about rate-limiting transition states and metastable poses that can in principle be used in the design of drugs with a desired kinetic profile. One important lesson from these simulations was that accurate rate estimates require consideration of ligand solvation, as also concluded by others (52, 65).

Recently, MetaD was also used to investigate the dissociation of a potent drug targeting the MOR, specifically fentanyl, from the receptor (54). Not only did these simulations provide information about fentanyl’s binding kinetics and mechanism, but they suggested a role for the protonation state of the conserved residue His^6.52^ in modulating fentanyl’s affinity and binding pose. While a recent study (76) reporting cryo-EM structures of the MOR bound to fentanyl showed that a His(6.52)Ala mutation reduced fentanyl-induced MOR activation of G protein and β-arrestin signaling, it did not support the predicted binding poses of fentanyl using MetaD or other computational methods (53, 54, 77).

Ligand binding kinetics is however not the only temporal aspect that is important to understand GPCR signaling. To tackle the kinetics of GPCR activation, we designed a computational strategy that uses a combination of PS-MetaD and the maximum caliber principle to not only thoroughly characterize the conformational space sampled by the receptor along its activation path, but also to derive unbiased kinetic rates from simulations with transitions accelerated by a bias potential (38) (**Figure 1D**). We showed that the approach could efficiently sample conformational transitions between the crystal structures of inactive and active GPCRs, specifically the MOR, at a ∼2 orders of magnitude computational cost reduction with respect to a more expensive high-throughput MD adaptive sampling protocol run on distributed computational resources. We also demonstrated that the strategy yielded thermodynamic and kinetic properties of MOR activation, as well as information about ligand-induced allosteric communications across the receptor when combined with n-body information theory (34), that were in agreement with those obtained by more computationally expensive adaptive sampling protocols.

## Conclusions

Identifying the molecular determinants that underlie the different dynamic and kinetic behaviors of GPCRs is likely to provide fundamental information for drug discovery, and is therefore the focus of much research. However, obtaining this information is not a trivial undertaking, either experimentally or computationally. While strategies combining different variants of MetaD with state-of-the-art algorithms for statistical analyses of simulation data and/or AI-based tools have made strides in the study of GPCR ligand binding, activation, and related kinetics, the majority of studies published to date are limited to the receptors alone, although recent studies are focusing more and more on receptor complexes and the combined effect of ligand and transducer on the dynamic behavior of the receptor. To better understand the underlying principles of GPCR biased signaling, attention will probably need to be shifted more towards the appropriate characterization of the dynamic behavior of interacting signaling proteins, thus providing important insight into receptor-transducer complex stability and/or receptor-transducer activation kinetics. These studies will most likely require developing more sophisticated MetaD strategies or hybrid approaches and a community effort to disseminate best practices for MetaD simulations, as well as system setups, parameters, protocols, etc. to more expeditiously advance knowledge of GPCR dynamics and their relation to functional selectivity.

## Author contributions

MF wrote the first draft of the manuscript. LS-E and BF made the table and figure, respectively. All authors contributed to the article and approved the submitted version.

## References

[1] Gurevich VV Gurevich EV. Molecular mechanisms of gpcr signaling: A structural perspective. Int J Mol Sci (2017) 18(12):2519. doi: 10.3390/ijms18122519 29186792 PMC5751122

[2] Bohn LM. Selectivity for G protein or arrestin-mediated signaling. In: K N, editor. Functional selectivity of G protein-coupled receptor ligands. Totowa, NJ: Humana Press (2009). p. 71–85.

[3] Kenakin T. New concepts in drug discovery: Collateral efficacy and permissive antagonism. Nat Rev Drug Discovery (2005) 4(11):919–27. doi: 10.1038/nrd1875 16264435

[4] Kenakin T. Collateral efficacy in drug discovery: Taking advantage of the good (Allosteric) nature of 7tm receptors. Trends Pharmacol Sci (2007) 28(8):407–15. doi: 10.1016/j.tips.2007.06.009 17629960

[5] Kenakin T. Biased agonism. F1000 Biol Rep (2009) 1:87. doi: 10.3410/B1-87 20948603 PMC2948287

[6] Mailman RB. Gpcr functional selectivity has therapeutic impact. Trends Pharmacol Sci (2007) 28(8):390–6. doi: 10.1016/j.tips.2007.06.002 PMC2958218 17629962

[7] Urban JD Clarke WP von Zastrow M Nichols DE Kobilka B Weinstein H. Functional selectivity and classical concepts of quantitative pharmacology. J Pharmacol Exp Ther (2007) 320(1):1–13. doi: 10.1124/jpet.106.104463 16803859

[8] Hoare SRJ Tewson PH Quinn AM Hughes TE Bridge LJ. Analyzing kinetic signaling data for G-Protein-Coupled receptors. Sci Rep (2020) 10(1):12263. doi: 10.1038/s41598-020-67844-3 32704081 PMC7378232

[9] Hoare SRJ Tewson PH Sachdev S Connor M Hughes TE Quinn AM. Quantifying the kinetics of signaling and arrestin recruitment by nervous system G-protein coupled receptors. Front Cell Neurosci (2021) 15:814547. doi: 10.3389/fncel.2021.814547 35110998 PMC8801586

[10] Swinney DC Haubrich BA Van Liefde I Vauquelin G. The role of binding kinetics in gpcr drug discovery. Curr Top Med Chem (2015) 15(24):2504–22. doi: 10.2174/1568026615666150701113054 26126905

[11] Wright SC Bouvier M. Illuminating the complexity of gpcr pathway selectivity - advances in biosensor development. Curr Opin Struct Biol (2021) 69:142–9. doi: 10.1016/j.sbi.2021.04.006 34048988

[12] Kooistra AJ Mordalski S Pandy-Szekeres G Esguerra M Mamyrbekov A Munk C. Gpcrdb in 2021: Integrating gpcr sequence, structure and function. Nucleic Acids Res (2021) 49(D1):D335–D43. doi: 10.1093/nar/gkaa1080 PMC7778909 33270898

[13] Berman HM Westbrook J Feng Z Gilliland G Bhat TN Weissig H. The protein data bank. Nucleic Acids Res (2000) 28(1):235–42. doi: 10.1093/nar/28.1.235 PMC102472 10592235

[14] Casiraghi M Point E Pozza A Moncoq K Baneres JL Catoire LJ. Nmr analysis of gpcr conformational landscapes and dynamics. Mol Cell Endocrinol (2019) 484:69–77. doi: 10.1016/j.mce.2018.12.019 30690069

[15] Cong X Maurel D Demene H Vasiliauskaite-Brooks I Hagelberger J Peysson F. Molecular insights into the biased signaling mechanism of the mu-opioid receptor. Mol Cell (2021) 81(20):4165–75 e6. doi: 10.1016/j.molcel.2021.07.033 34433090 PMC8541911

[16] Eddy MT Lee MY Gao ZG White KL Didenko T Horst R. Allosteric coupling of drug binding and intracellular signaling in the A2a adenosine receptor. Cell (2018) 172(1-2):68–80 e12. doi: 10.1016/j.cell.2017.12.004 29290469 PMC5766378

[17] Huang SK Pandey A Tran DP Villanueva NL Kitao A Sunahara RK. Delineating the conformational landscape of the adenosine A2a receptor during G protein coupling. Cell (2021) 184(7):1884–94 e14. doi: 10.1016/j.cell.2021.02.041 33743210 PMC10266235

[18] Okude J Ueda T Kofuku Y Sato M Nobuyama N Kondo K. Identification of a conformational equilibrium that determines the efficacy and functional selectivity of the mu-opioid receptor. Angew Chem Int Ed Engl (2015) 54(52):15771–6. doi: 10.1002/anie.201508794 PMC4722849 26568421

[19] Picard LP Prosser RS. Advances in the study of gpcrs by (19)F nmr. Curr Opin Struct Biol (2021) 69:169–76. doi: 10.1016/j.sbi.2021.05.001 34130235

[20] Sounier R Mas C Steyaert J Laeremans T Manglik A Huang W. Propagation of conformational changes during mu-opioid receptor activation. Nature (2015) 524(7565):375–8. doi: 10.1038/nature14680 PMC4820006 26245377

[21] Xu J Hu Y Kaindl J Risel P Hubner H Maeda S. Conformational complexity and dynamics in a muscarinic receptor revealed by nmr spectroscopy. Mol Cell (2019) 75(1):53–65 e7. doi: 10.1016/j.molcel.2019.04.028 31103421

[22] Elgeti M Hubbell WL. Deer analysis of gpcr conformational heterogeneity. Biomolecules (2021) 11(6):778. doi: 10.3390/biom11060778 34067265 PMC8224605

[23] Du Y Duc NM Rasmussen SGF Hilger D Kubiak X Wang L. Assembly of a gpcr-G protein complex. Cell (2019) 177(5):1232–42 e11. doi: 10.1016/j.cell.2019.04.022 31080064 PMC6763313

[24] Liu X Xu X Hilger D Aschauer P Tiemann JKS Du Y. Structural insights into the process of gpcr-G protein complex formation. Cell (2019) 177(5):1243–51 e12. doi: 10.1016/j.cell.2019.04.021 31080070 PMC6991123

[25] Asher WB Terry DS Gregorio GGA Kahsai AW Borgia A Xie B. Gpcr-mediated beta-arrestin activation deconvoluted with single-molecule precision. Cell (2022) 185(10):1661–75 e16. doi: 10.1016/j.cell.2022.03.042 35483373 PMC9191627

[26] Kauk M Hoffmann C. Intramolecular and intermolecular fret sensors for gpcrs - monitoring conformational changes and beyond. Trends Pharmacol Sci (2018) 39(2):123–35. doi: 10.1016/j.tips.2017.10.011 29180026

[27] Quast RB Margeat E. Studying gpcr conformational dynamics by single molecule fluorescence. Mol Cell Endocrinol (2019) 493:110469. doi: 10.1016/j.mce.2019.110469 31163201

[28] Latorraca NR Venkatakrishnan AJ Dror RO. Gpcr dynamics: Structures in motion. Chem Rev (2017) 117(1):139–55. doi: 10.1021/acs.chemrev.6b00177 27622975

[29] Abrol R Serrano E Santiago LJ. Development of enhanced conformational sampling methods to probe the activation landscape of gpcrs. Adv Protein Chem Struct Biol (2022) 128:325–59. doi: 10.1016/bs.apcsb.2021.11.001 PMC11476118 35034722

[30] Laio A Parrinello M. Escaping free-energy minima. P Natl Acad Sci USA (2002) 99(20):12562–6. doi: 10.1073/pnas.202427399 PMC130499 12271136

[31] Provasi D Bortolato A Filizola M. Exploring molecular mechanisms of ligand recognition by opioid receptors with metadynamics. Biochemistry (2009) 48(42):10020–9. doi: 10.1021/bi901494n PMC2764813 19785461

[32] Pérez-Hernández G Paul F Giorgino T De Fabritiis G Noé F. Identification of slow molecular order parameters for Markov model construction. J Chem Phys (2013) 139:15102. doi: 10.1063/1.4811489 23822324

[33] Schwantes CR Pande VS. Improvements in Markov state model construction reveal many non-native interactions in the folding of Ntl9. J Chem Theory Comput (2013) 9(4):2000–9. doi: 10.1021/ct300878a PMC3673732 23750122

[34] LeVine MV Weinstein H. Nbit–a new information theory-based analysis of allosteric mechanisms reveals residues that underlie function in the leucine transporter leut. PloS Comput Biol (2014) 10(5):e1003603. doi: 10.1371/journal.pcbi.1003603 24785005 PMC4006702

[35] Schreiber T. Measuring information transfer. Phys Rev Lett (2000) 85:461. doi: 10.1103/PhysRevLett.85.461 10991308

[36] Noe F Tkatchenko A Muller KR Clementi C. Machine learning for molecular simulation. Annu Rev Phys Chem (2020) 71:361–90. doi: 10.1146/annurev-physchem-042018-052331 32092281

[37] Lamim Ribeiro JM Provasi D Filizola M. A combination of machine learning and infrequent metadynamics to efficiently predict kinetic rates, transition states, and molecular determinants of drug dissociation from G protein-coupled receptors. J Chem Phys (2020) 153(12):124105. doi: 10.1063/5.0019100 33003748 PMC7515652

[38] Meral D Provasi D Filizola M. An efficient strategy to estimate thermodynamics and kinetics of G protein-coupled receptor activation using metadynamics and maximum caliber. J Chem Phys (2018) 149(22):224101. doi: 10.1063/1.5060960 30553249 PMC6291190

[39] Saleh N Ibrahim P Saladino G Gervasio FL Clark T. An efficient metadynamics-based protocol to model the binding affinity and the transition state ensemble of G-Protein-Coupled receptor ligands. J Chem Inf Model (2017) 57(5):1210–7. doi: 10.1021/acs.jcim.6b00772 28453271

[40] Soldner CA Horn AHC Sticht H. A metadynamics-based protocol for the determination of gpcr-ligand binding modes. Int J Mol Sci (2019) 20(8):1970. doi: 10.3390/ijms20081970 31013635 PMC6514967

[41] Shang Y Yeatman HR Provasi D Alt A Christopoulos A Canals M. Proposed mode of binding and action of positive allosteric modulators at opioid receptors. ACS Chem Biol (2016) 11(5):1220–9. doi: 10.1021/acschembio.5b00712 PMC4950826 26841170

[42] Crowley RS Riley AP Sherwood AM Groer CE Shivaperumal N Biscaia M. Synthetic studies of neoclerodane diterpenes from salvia divinorum: Identification of a potent and centrally acting mu opioid analgesic with reduced abuse liability. J Med Chem (2016) 59(24):11027–38. doi: 10.1021/acs.jmedchem.6b01235 PMC5189922 27958743

[43] Zhou Y Ramsey S Provasi D El Daibani A Appourchaux K Chakraborty S. Predicted mode of binding to and allosteric modulation of the mu-opioid receptor by kratom's alkaloids with reported antinociception in vivo. Biochemistry (2021) 60(18):1420–9. doi: 10.1021/acs.biochem.0c00658 PMC8119294 33274929

[44] Kapoor A Provasi D Filizola M. Atomic-level characterization of the methadone-stabilized active conformation of mu-opioid receptor. Mol Pharmacol (2020) 98(4):475–86. doi: 10.1124/mol.119.119339 PMC7562981 32680919

[45] Mafi A Kim SK Goddard WA 3rd. The mechanism for ligand activation of the gpcr-G protein complex. Proc Natl Acad Sci U.S.A. (2022) 119(18):e2110085119. doi: 10.1073/pnas.2110085119 35452328 PMC9170043

[46] Provasi D Artacho MC Negri A Mobarec JC Filizola M. Ligand-induced modulation of the free-energy landscape of G protein-coupled receptors explored by adaptive biasing techniques. PloS Comput Biol (2011) 7(10):e1002193. doi: 10.1371/journal.pcbi.1002193 22022248 PMC3192824

[47] Saleh N Saladino G Gervasio FL Clark T. Investigating allosteric effects on the functional dynamics of Beta2-adrenergic ternary complexes with enhanced-sampling simulations. Chem Sci (2017) 8(5):4019–26. doi: 10.1039/c6sc04647a PMC6094175 30155211

[48] Li J Jonsson AL Beuming T Shelley JC Voth GA. Ligand-dependent activation and deactivation of the human adenosine a(2a) receptor. J Am Chem Soc (2013) 135(23):8749–59. doi: 10.1021/ja404391q PMC4120839 23678995

[49] Yang MY Kim SK Goddard WA 3rd. G Protein coupling and activation of the metabotropic gabab heterodimer. Nat Commun (2022) 13(1):4612. doi: 10.1038/s41467-022-32213-3 35941188 PMC9360005

[50] Mattedi G Acosta-Gutierrez S Clark T Gervasio FL. A combined activation mechanism for the glucagon receptor. Proc Natl Acad Sci U.S.A. (2020) 117(27):15414–22. doi: 10.1073/pnas.1921851117 PMC7355025 32571939

[51] Provasi D Filizola M. Putative active states of a prototypic G-Protein-Coupled receptor from biased molecular dynamics. Biophys J (2010) 98(10):2347–55. doi: 10.1016/j.bpj.2010.01.047 PMC2872269 20483344

[52] Deganutti G Zhukov A Deflorian F Federico S Spalluto G Cooke RM. Impact of protein-ligand solvation and desolvation on transition state thermodynamic properties of adenosine A2a ligand binding kinetics. In Silico Pharmacol (2017) 5(1):16. doi: 10.1007/s40203-017-0037-x 29308352 PMC5755719

[53] Vo QN Mahinthichaichan P Shen J Ellis CR. How mu-opioid receptor recognizes fentanyl. Nat Commun (2021) 12(1):984. doi: 10.1038/s41467-021-21262-9 33579956 PMC7881245

[54] Mahinthichaichan P Vo QN Ellis CR Shen J. Kinetics and mechanism of fentanyl dissociation from the mu-opioid receptor. JACS Au (2021) 1(12):2208–15. doi: 10.1021/jacsau.1c00341 PMC8715493 34977892

[55] Valsson O Tiwary P Parrinello M. Enhancing important fluctuations: Rare events and metadynamics from a conceptual viewpoint. Annu Rev Phys Chem (2016) 67:159–84. doi: 10.1146/annurev-physchem-040215-112229 26980304

[56] Barducci A Bussi G Parrinello M. Well-tempered metadynamics: A smoothly converging and tunable free-energy method. Phys Rev Lett (2008) 100(2):20603. doi: 10.1103/PhysRevLett.100.020603 18232845

[57] Allen TW Andersen OS Roux B. Energetics of ion conduction through the gramicidin channel. Proc Natl Acad Sci U.S.A. (2004) 101(1):117–22. doi: 10.1073/pnas.2635314100 PMC314148 14691245

[58] Schlechtingen G DeHaven RN Daubert JD Cassel JA Chung NN Schiller PW. Structure-activity relationships of dynorphin a analogues modified in the address sequence. J Med Chem (2003) 46(11):2104–9. doi: 10.1021/jm020125+ 12747782

[59] Toll L Berzetei-Gurske IP Polgar WE Brandt SR Adapa ID Rodriguez L. Standard binding and functional assays related to medications development division testing for potential cocaine and opiate narcotic treatment medications. NIDA Res Monogr (1998) 178:440–66. 9686407

[60] Tryoen-Toth P Decaillot FM Filliol D Befort K Lazarus LH Schiller PW. Inverse agonism and neutral antagonism at wild-type and constitutively active mutant delta opioid receptors. J Pharmacol Exp Ther (2005) 313(1):410–21. doi: 10.1124/jpet.104.077321 15590769

[61] Valenzano KJ Miller W Chen Z Shan S Crumley G Victory SF. Dipoa ([8-(3,3-Diphenyl-Propyl)-4-Oxo-1-Phenyl-1,3,8-Triazaspiro[4.5]Dec-3-Yl]-Acetic acid), a novel, systemically available, and peripherally restricted mu opioid agonist with antihyperalgesic activity: I. *In vitro* pharmacological characterization and pharmacokinetic properties. J Pharmacol Exp Ther (2004) 310(2):783–92. doi: 10.1124/jpet.103.063313 15054115

[62] Ibrahim P Clark T. Metadynamics simulations of ligand binding to gpcrs. Curr Opin Struct Biol (2019) 55:129–37. doi: 10.1016/j.sbi.2019.04.002 31100549

[63] Schneider S Provasi D Filizola M. The dynamic process of drug-gpcr binding at either orthosteric or allosteric sites evaluated by metadynamics. Methods Mol Biol (2015) 1335:277–94. doi: 10.1007/978-1-4939-2914-6_18 PMC4703114 26260607

[64] Ballesteros JA Weinstein H. Integrated methods for the construction of three-dimensional models and computational probing of structure-function relations in G protein-coupled receptors. Methods Neurosci (1995) 25:366–428. doi: 10.1016/S1043-9471(05)80049-7

[65] Limongelli V Marinelli L Cosconati S La Motta C Sartini S Mugnaini L. Sampling protein motion and solvent effect during ligand binding. Proc Natl Acad Sci U.S.A. (2012) 109(5):1467–72. doi: 10.1073/pnas.1112181108 PMC3277130 22238423

[66] Raniolo S Limongelli V. Ligand binding free-energy calculations with funnel metadynamics. Nat Protoc (2020) 15(9):2837–66. doi: 10.1038/s41596-020-0342-4 32814837

[67] Clark AJ Tiwary P Borrelli K Feng S Miller EB Abel R. Prediction of protein-ligand binding poses *Via* a combination of induced fit docking and metadynamics simulations. J Chem Theory Comput (2016) 12(6):2990–8. doi: 10.1021/acs.jctc.6b00201 27145262

[68] Dror RO Arlow DH Maragakis P Mildorf TJ Pan AC Xu H. Activation mechanism of the Beta2-adrenergic receptor. Proc Natl Acad Sci U.S.A. (2011) 108(46):18684–9. doi: 10.1073/pnas.1110499108 PMC3219117 22031696

[69] Bussi G Gervasio FL Laio A Parrinello M. Free-energy landscape for beta hairpin folding from combined parallel tempering and metadynamics. J Am Chem Soc (2006) 128(41):13435–41. doi: 10.1021/ja062463w 17031956

[70] Deighan M Bonomi M Pfaendtner J. Efficient simulation of explicitly solvated proteins in the well-tempered ensemble. J Chem Theory Comput (2012) 8(7):2189–92. doi: 10.1021/ct300297t 26588950

[71] Dror RO Pan AC Arlow DH Borhani DW Maragakis P Shan Y. Pathway and mechanism of drug binding to G-Protein-Coupled receptors. Proc Natl Acad Sci U.S.A. (2011) 108(32):13118–23. doi: 10.1073/pnas.1104614108 PMC3156183 21778406

[72] Ravindra P Smith Z Tiwary P. Automatic mutual information noise omission (Amino): Generating order parameters for molecular systems. Mol Syst Des Eng (2020) 5:339–48. doi: 10.1039/C9ME00115H

[73] Wang Y Ribeiro JML Tiwary P. Past-future information bottleneck for sampling molecular reaction coordinate simultaneously with thermodynamics and kinetics. Nat Commun (2019) 10(1):3573. doi: 10.1038/s41467-019-11405-4 31395868 PMC6687748

[74] Wang Y Tiwary P. Understanding the role of predictive time delay and biased propagator in rave. J Chem Phys (2020) 152(14):144102. doi: 10.1063/5.0004838 32295373

[75] Pedersen MF Wrobel TM Marcher-Rorsted E Pedersen DS Moller TC Gabriele F. Biased agonism of clinically approved mu-opioid receptor agonists and Trv130 is not controlled by binding and signaling kinetics. Neuropharmacology (2020) 166:107718. doi: 10.1016/j.neuropharm.2019.107718 31351108

[76] Zhuang Y Wang Y He B He X Zhou XE Guo S. Molecular recognition of morphine and fentanyl by the human M-opioid receptor. Cell (2022) 185(23):4361–75.e19. doi: 10.1016/j.cell.2022.09.041 36368306

[77] Ricarte A Dalton JAR Giraldo J. Structural assessment of agonist efficacy in the mu-opioid receptor: Morphine and fentanyl elicit different activation patterns. J Chem Inf Model (2021) 61(3):1251–74. doi: 10.1021/acs.jcim.0c00890 33448226

